# Analytical verification and comparison of chromogenic assays for dabigatran, rivaroxaban and apixaban determination on BCSXP and STA Compact Max analysers

**DOI:** 10.11613/BM.2020.010706

**Published:** 2020-02-15

**Authors:** Ivana Ćelap, Sandra Margetić, Marija Brčić, Roman Mihić

**Affiliations:** 1 Department of Clinical Chemistry, Sestre milosrdnice University Hospital Center, Zagreb, Croatia

**Keywords:** chromogenic assay, dabigatran, rivaroxaban, apixaban, method comparison

## Abstract

**Introduction:**

The aim of the study was to perform analytical verification and comparison of chromogenic assays for determination of dabigatran, rivaroxaban and apixaban concentration on BCSXP and STA Compact Max analysers.

**Materials and methods:**

Precision, linearity, measurement uncertainty estimation and determination of limit of blank, limit of determination and limit of quantification were calculated. Analytical performance specifications were set according to manufacturer specifications and literature data on between laboratory variability. Comparison of the methods was done using Bland-Altman and Passing-Bablok regression analysis.

**Results:**

Obtained results have shown acceptable precision on STA Compact Max only for dabigatran (CV = 3.5%) at lower concentration level comparing to manufacturer declaration (CV = 3.6%). On BCSXP, the highest coefficient of variation has been shown for apixaban (6.1%) at lower concentration level. Within laboratory precision was not met on STA Compact Max for all assays. Bland-Altman analysis has shown statistically significant bias for dabigatran (23.2%, 95%CI 11.2 – 35.3; P < 0.001) and apixaban (8.4%, 95%CI 1.2 – 15.6; P = 0.023). Passing-Bablok regression analysis has shown systematic and proportional deviation between methods for rivaroxaban (y = 6.52 (2.94 to 11.83) + 0.84 (0.80 to 0.89) x.

**Conclusion:**

Chromogenic assays for dabigatran, rivaroxaban and apixaban on BCSXP and STA Compact Max analysers are shown as methods with satisfactory long-term analytical performance specifications for determination of direct oral anticoagulants in clinical laboratories. However, we cannot recommend interchangeable use because of the significant bias between assays.

## Introduction

Although designed as a *one-size-fits all* drugs, the fixed-dose approach in treatment with direct oral anticoagulants (DOACs) has its weaknesses. Today, high intra-individual and inter-individual variability in drug response has been shown, depending on different factors (*1*). Thus, it is recommended to measure DOAC concentration in blood in certain clinical situations. Current guidelines do not recommend routine monitoring of DOAC concentrations but sporadic measuring in specific cases such as an emergency surgical operation, sudden extensive bleeding, thromboembolic events, *etc.* (*2*). Since global coagulation tests have been shown as non-specific for the evaluation of the anticoagulant effect of DOACs, specific assays have been developed with the accent on liquid chromatography coupled with tandem mass spectrometry method (LC-MS/MS) as the most accurate for the determination of DOAC concentration (*3*-*5*). However, the complexity of the LC-MS/MS method requires highly trained personnel and expensive equipment limiting its availability. One of the most important factors in determination of the DOAC concentration, in recommended situations, is promptness in obtaining the results. To bridge the issues with LC-MS/MS, coagulation assays manufacturers developed functional assays for determination of DOACs concentration remaining LC-MS/MS as a gold standard. Looking overall, DOAC specific coagulation assays have some benefits over mass spectrometry in terms of affordability, easiness of use and shorter turnaround time (TAT) (*2*). To the best of our knowledge, there are no published data on analytical performance (*e.g.* precision study; total laboratory variability; comparison of the patient results between two or more analysers) of these assays. Thus, the aim of the study was to perform analytical verification and comparison of chromogenic assays for determination of dabigatran, rivaroxaban and apixaban concentration on BCSXP and STA Compact Max analysers.

## Material and methods

### Study design

The study was performed at the Department of Clinical Chemistry, Sestre milosrdnice University Hospital Center in September 2017 as a part of the project IP-2016-06-8208 funded by the Croatian Scientific Foundation. Analytical verification for all DOAC assays, on both analysers, included repeatability, intermediate precision and within laboratory precision, linearity and method comparison as recommended by the International Council for Standardization in Haematology (ICSH). The study was approved by Sestre milosrdnice University Hospital Center Ethic committee.

### Methods

Citrate plasma samples (N = 138) for linearity studies (N = 6), method comparison (N = 120), determination of limit of detection (N = 6) and limit of quantification (N = 6) have been obtained from the patients who are treated with the one of the DOACs (dabigatran, rivaroxaban and apixaban) and who participate in the IP-2016-06-8208 project. For the determination of limit of blank, leftover citrate plasma samples (N = 3) from subjects who are not using DOACs or any other anticoagulation drug has been used. Venous blood was collected in 3.2% sodium citrate containing vacutainers (Greiner Bio-One, Kremsmünster, Austria) and centrifuged within one hour after blood drawing at 3500xg for 10 minutes.

Concentrations of dabigatran, rivaroxaban and apixaban were determined on coagulation analysers BCSXP (Siemens Healthineers, Marburg, Germany) and STA Compact Max (Diagnostica Stago, Asnieres sur Seine, France).

On BCSXP analyser, dabigatran concentrations were measured using Innovance DTI assay (Siemens Healthineers, Marburg, Germany) calibrated with Dabigatran Standards (Ref. No. OPOL93) (Siemens Healthineers, Marburg, Germany). Rivaroxaban and apixaban were determined using Innovance Heparin (Siemens Healthineers, Marburg, Germany) calibrated with BIOPHEN™ Rivaroxaban Calibrator Low (Ref. No. 226001), BIOPHEN™ Rivaroxaban Calibrator (Ref. No. 222701), BIOPHEN™ Apixaban Calibrator Low (Ref. No. 226101) and BIOPHEN™ Apixaban Calibrator (Ref. No. 226201), respectively. All BIOPHEN™ calibrators are products of HYPHEN BioMed (Neuville-sur-Oise, France).

On STA Compact Max, dabigatran was measured using STA-ECA II (Ref. No. 00992) (Diagnostica Stago, Asnieres sur Seine, France) calibrated with STA-Dabigatran Calibrator (Ref. No. 00993) (Diagnostica Stago, Asnieres sur Seine, France). Rivaroxaban and apixaban were determined using STA-Liquid Anti-Xa assay (Ref. No. 00311) (Diagnostica Stago, Asnieres sur Seine, France) calibrated with STA-Rivaroxaban Calibrator (Ref. No. 00704) (Diagnostica Stago, Asnieres sur Seine, France) and STA-Apixaban Calibrator (Ref. No. 01075) (Diagnostica Stago, Asnieres sur Seine, France), respectively.

The manufacturers’ protocol for each assay was used on both automated coagulation systems.

### Precision studies

Repeatability, intermediate and within laboratory precision were calculated using two levels (low and high concentration) of control plasma samples for each DOAC on both, BCSXP and STA Compact Max analysers. On BCSXP analyser following controls were used: i) Dabigatran controls (Ref. No. OPOK03) (Siemens Healthineers, Marburg, Germany); ii) BIOPHEN™ Rivaroxaban Control Plasma (Ref. No. 224501); iii) BIOPHEN™ Rivaroxaban Control Low (Ref. No. 225101); iv) BIOPHEN™ Apixaban Control Plasma (Ref. No. 225301) and v) BIOPHEN™ Apixaban Control Low (Ref. No. 225201). All Biophen controls are products of HYPHEN BioMed (Neuville-sur-Oise, France).

For precision studies on Stago Compact Max original controls were used: i) Dabigatran control (Ref. No. 00994); ii) Rivaroxaban control (Ref. No. 00706) and iii) Apixaban control (Ref. No. 01074), all products of Diagnostica Stago (Asnieres sur Seine, France). Each level of control samples, for all DOAC assays, was measured in triplicate during five days and coefficients of variation (CVs) were calculated according to CLSI EP15 A2. Afterwards, obtained CVs were compared with manufacturers’ precision limits for each DOAC.

### Linearity

Linearity for each assay, on both analysers, was checked using one patient sample pool in five concentration points. For each DOAC one sample with the concentration of DOAC near upper level (H) of measurement range was serially diluted (H; 1:3; 1:1; 3:1; L) with one sample with the concentration of DOAC near lower level (L) of the declared measurement range (Table 1), according to CLSI E6-A. Acceptable bias between expected and measured value were 20%.

**Table 1:** Manufacturer’s declarations on measurement range

**Analyser**	**Assay**	**Measurement range (ng/mL)**
	Dabigatran	20 – 500
**BCSXP**	Rivaroxaban	20 – 500
	Apixaban	< 500
	Dabigatran	15 – 460
**STA Compact Max**	Rivaroxaban	25 – 500
	Apixaban	23 – 500

### Measurement uncertainty

Initial expanded measurement uncertainty (MU) was calculated for all tests from the within laboratory precision (*S_l_*) multiplied with the coverage factor (k = 2) using the following calculation: U = *k* × *S*_l_. Acceptance criteria for MU was arbitrarily defined as less than 20%.

### Method comparison

Comparison of DOAC concentrations between STA Compact Max and BCSXP was done by determination of DOAC concentrations in 40 patient samples at the same time on both analysers, covering reportable ranges for all three DOACs.

### Limit of blank, limit of detection and limit of quantification

Leftover plasma sample from patients not treated with DOACs was tested twelve times in a batch analysis to obtain limit of blank (LoB). Obtained values were compared with manufacturers’ declaration, if given. Limit of detection (LoD) was determined by testing patient plasma samples, with the concentration of DOAC near the detection limit claimed by manufacturers, twenty times in a batch analysis. Concentration at which 95% results were below manufacturers’ LoD declaration was considered as LoD.

As for the limit of quantification (LoQ), patient plasma samples, with the concentration of DOACs near the declared LoQ by the manufacturers, if given, were tested twenty times in a batch analysis. Concentration at which less than 5% of results had bias between first and every other measurement below 5% was considered as LoQ.

### Statistical analysis

For precision analysis, mean, standard deviation and coefficient of variation was calculated. Method comparison was done using Passing-Bablok and Bland-Altman analysis. Limit of blank, LoD and LoQ were calculated using Microsoft Excel version 2010 (Microsoft Corporation, Redmond Washington) and calculations given by Armbruster and Pry (*7*). A P < 0.05 was set as statistically significant. Statistical analysis was performed using Medcalc statistical software version 19.0.3 (Medcalc Statistical Software, Ostend, Belgium).

## Results

Precision studies and measurement uncertainty estimation for dabigatran, rivaroxaban, and apixaban on BCSXP analyser are presented in Table 2. Repeatability and within laboratory precision showed low coefficient of variation but only for dabigatran manufacturer criteria are met. As presented in Table 3, STA Compact Max precision study results have shown acceptable performance according to previously defined criteria only for within laboratory precision (*S_l_*). Table 4 presents the obtained concentrations for LoB, LoD and LoQ for each drug and on both analysers. Results have shown that obtained LoD concentrations were lower than that declared by the manufacturer. Furthermore, on both analysers, LoB for dabigatran was 0 ng/mL whereas rivaroxaban and apixaban LoB concentrations were higher on STA Compact Max than on BCSXP.

**Table 2:** Precision studies on BCSXP analyser

	**Dabigatran**		**Rivaroxaban**		**Apixaban**	
	**L 1**	**L 2**	**L 1**	**L 2**	**L 1**	**L 2**
**X_sr_**	82.6	249.3	26.2	337.1	26.4	426.8
**S_r_**	2.59	3.34	1.00	3.78	1.61	6.76
**CV%**	3.14	1.34	3.81	1.12	6.11	1.58
**Acceptance criteria (CV%, manufacturer)**	4.01	1.42	/*	/*	/*	/*
**S_l_**	5.77	6.39	1.32	7.81	1.58	8.97
**CV%**	6.99	2.57	5.06	2.32	5.99	2.10
**Acceptance criteria (CV%, manufacturer)**	10.00	5.00	/*	/*	/*	/*
**U (k = 2)**	14	5	10	5	12	4
**Acceptance criteria^†^ (%)**	20	20	20	20	20	20
L – control level. X_sr_ – arithmetic mean. S_r_ – repeatability. CV – coefficient of variation. S_I_ –within laboratory precision. U – expanded measurement uncertainty. *Manufacturer does not declare CVs for combination of Biophen control plasma and calibrator for rivaroxaban and apixaban and Innovance Heparin assay. ^†^Arbitrarily defined criteria using published data on analytical variability of the methods according to van Colt *et al.* (6).						

**Table 3:** Precision studies on STA Compact Max analyser

	**Dabigatran**		**Rivaroxaban**		**Apixaban**		
	**L 1**	**L 2**	**L 1**	**L 2**	**L 1**	**L 2**	
**X_sr_**	55.1	202.4	86.7	323.0	75.2	277.6	
**S_r_**	1.92	4.58	2.36	6.32	2.47	6.81	
**CV%**	3.50	2.26	2.73	1.96	3.29	2.45	
**Acceptance criteria (CV%, manufacturer)**	3.60	2.00	2.50	1.90	2.80	2.00	
**S_l_**	2.13	3.89	2.74	8.49	5.36	9.12	
**CV%**	3.86	1.92	3.16	2.63	7.12	3.29	
**Acceptance criteria (CV%, manufacturer)**	5.20	3.10	3.30	2.80	6.00	4.40	
**U (k = 2)**	8	4	6	5	14	7	
Acceptance criteria* (%)	20	20	20	20	20	20	
L – control level. X_sr_ – arithmetic mean. S_r_ – repeatability. CV – coefficient of variation. S_l_ – within laboratory precision. U – expanded measurement uncertainty. *Arbitrarily defined criteria using publish data on analytical variability of the methods according to van Colt *et al.* (6)							

**Table 4:** Obtained values for limit of blank, limit of detection and limit of quantification

		**LoB (ng/mL)**		**LoD (ng/mL)**		**LoQ (ng/mL)**	
		**Laboratory**	**Manufacturer**	**Laboratory**	**Manufacturer**	**Laboratory**	**Manufacturer**
	dabigatran	0	/	2.8	14.8	34	20
**BCSXP**	rivaroxaban	3.89	/	5.9	/	24	/
	apixaban	2.72	/	4.1	/	27	/
**STA**	dabigatran	0	/	4.3	15.0	20	/
**Compact**	rivaroxaban	11.1	/	13.6	25.0	34	/
**Max**	apixaban	12.3	/	15.8	20.0	25	/
LoB – limit of blank. LoD – limit of detection. LoQ – limit of quantification. “/” - data not given.							

Linearity study has shown satisfactory biases between expected and obtained values for all DOACs. However, higher biases could be observed at the lower concentrations of measurement ranges. The highest bias (20%) was obtained for dabigatran at 20 ng/mL and apixaban (18.8%) at 48 ng/mL on STA Compact Max (data not shown).

**Figure 1:**
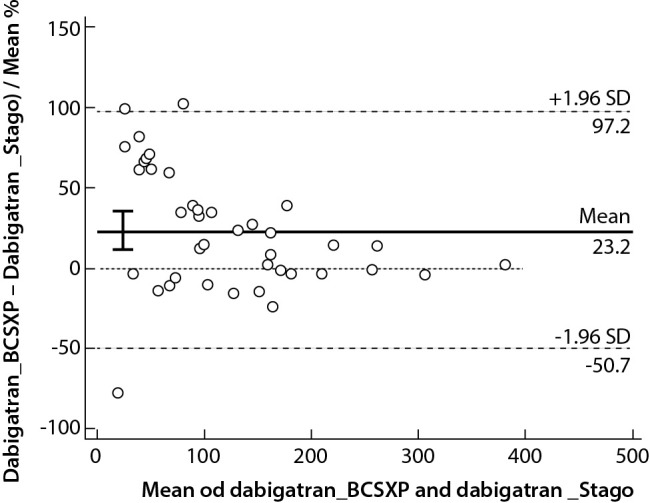
Relative bias between mean values of dabigatran measured on BCSXP and Stago Compact Max analyser using Bland-Altman analysis. Graph shows statistically significant bias between methods with the mean bias of 23.2% (continuous line). Vertical line represents confidence interval of the relative mean bias (11.18 to 35.31). SD – standard deviation (dashed lines).

**Figure 2:**
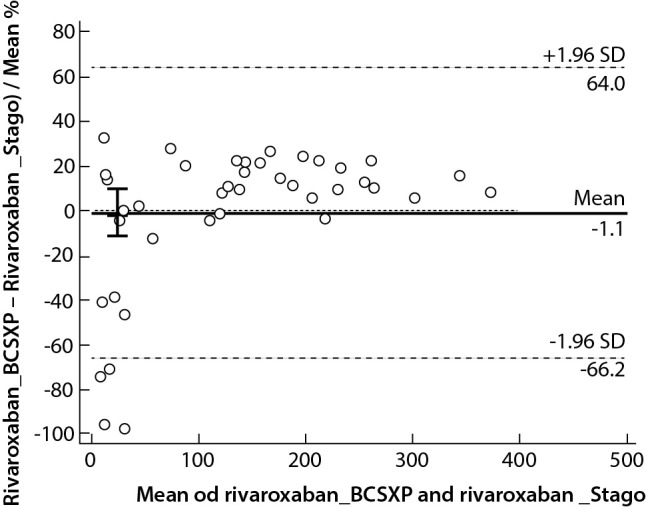
Relative bias between mean values of rivaroxaban measured on BCSXP and Stago Compact Max analyser using Bland-Altman analysis. Graph shows there is no statistically significant bias between methods with the mean bias of - 1.1% (continuous line). Vertical line represents confidence interval of the relative mean bias (-11.70 to 9.53). SD – standard deviation (dashed lines).

**Figure 3:**
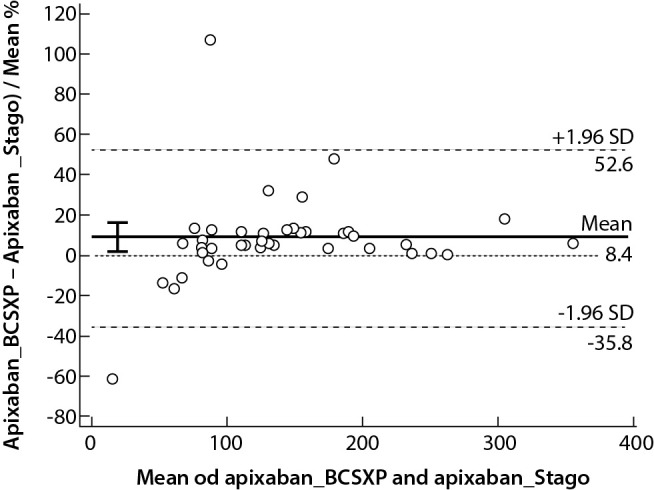
Relative bias between mean values of apixaban measured on BCSXP and Stago Compact Max analyser using Bland-Altman analysis. Graph shows statistically significant bias between methods with the mean bias of 8.4% (continuous line). Vertical line represents confidence interval of the relative mean bias (1.18 to 15.61). SD – standard deviation (dashed lines).

Finally, we have done a comparison of 40 patient samples for each DOAC on both coagulation analysers. Bland Altman analysis has shown statistically significant bias for dabigatran (23.2%, 95%CI 11.18 – 35.3; P < 0.001) and apixaban (8.4%, 95%CI 1.18 – 15.61; P = 0.023), while there was no significant bias for rivaroxaban (1.1%, 95%CI -11.70 – 9.53; P = 0.837) (Figures 1-3). Passing Bablok regression analysis has shown systematic and proportional deviation for rivaroxaban, the systematic deviation for dabigatran and proportional deviation for apixaban (Table 5).

**Table 5:** Passing Bablok regression analysis

	**N**	**BCSXP** **mean conc. (ng/mL)**	**STA Compact Max.** **mean conc.** **(ng/mL)**	**Intercept** **95%CI**	**Slope** **95%CI**	**P** **(Cusum test)**
Dabigatran	40	129.5 ± 81.3	112.5 ± 85.6	- 40.73 to -18.69	0.95 to 1.17	0.150
Rivaroxaban	40	139.8 ± 109.9	125.0 ± 93.8	2.94 to 11.83	0.80 to 0.89	0.970
Apixaban	40	133.5 ± 76.1	121.5 ± 69.8	- 0.85 to 13.37	0.83 to 0.96	0.300

## Discussion

Obtained results have not shown satisfactory repeatability for all assays on STA Compact Max when compared with manufacturer declaration. However, within laboratory precision and measurement uncertainty fulfil established criteria. Results of repeatability for dabigatran on BCSXP analyser have shown satisfactory analytical performance. Precision study results for rivaroxaban and apixaban cannot be evaluated according to manufacturer declarations since analytical performance specifications for these assays have not been provided. Namely, the determination of rivaroxaban and apixaban concentrations on BCSXP analyser is a modification of anti-FXa assay because it is calibrated with rivaroxaban and apixaban standards from a different manufacturer. However, obtained results showed satisfactory precision if compared with specifications given for Innovance Heparin reagent.

As for precision, information for LoB, LoD, and LoQ are partially lacking in manufacturers’ package inserts. Stago reagents declarations give only information on LoD, while Siemens provide LoD and LoQ for dabigatran. Our results revealed different LoQs between analysers for all three drugs. Limits of quantification for rivaroxaban and apixaban obtained on BCSXP analyser are sufficient for the determination of clinically relevant drug concentrations (> 30 ng/mL) (*2*). On the other hand, LoQ for rivaroxaban on STA Compact Max analyser exceeds that limit. Despite lower repeatability, coefficient of variation for rivaroxaban on STA Compact Max analyser at lower concentrations, higher LoB and LoD could be the reason for higher LoQ. Expected values given in guidelines for trough concentrations for all DOACs fall far below obtained LoQ values, but one must be aware of the fact that we set LoQ at the value with 5% CV (*2*). Although measurement ranges for all assays allow measuring lower values, caution have to be taken especially at concentrations below 20 ng/mL since all methods have shown a variability of more than 20%. Our results have confirmed previous studies that emphasized the importance of measuring exact DOAC concentration at low levels by LC-MS/MS (*5*).

Comparison of patient results for DOAC concentrations obtained on two analysers revealed significant differences in results for dabigatran and apixaban, whereas for rivaroxaban bias was not detected. However, it could be observed that concentrations of rivaroxaban lower than 50 ng/mL show a trend for higher bias. The study of the Italian external quality assessment scheme results on DOACs performance have shown very high variability in results obtained in plasma samples free from DOACs whereas variation at relatively low concentrations of rivaroxaban (81 ng/mL) and apixaban (66 ng/mL) were 8.4% and 10.3%, respectively (*8*). Further, the study by Van Cott *et al.* revealed poorer inter-laboratory precision for rivaroxaban at concentrations of 100 ng/mL than that at 300 ng/mL (*6*). Unfortunately, unlike Tripodi *et al.* the authors did not provide variability of the results obtained with different methods, but the differences between mean values obtained with different reagents/calibrators could be noticed (*6*, *8*). Recently, Hollestelle and Meijer have reported results of comparability from 10 surveys of external quality assessment, which revealed significant differences in CVs between reagents and/or manufacturers for rivaroxaban at lower concentrations (< 100 ng/mL) (*9*). Considering all of the above, we have set an allowable limit for MU and bias between instruments (< 20%).

Additionally, to recommendations for DOAC assay verification, we have estimated initial expanded measurement uncertainty. Measurement uncertainty is still not widely incorporated in analytical verification studies, but in our opinion, MU estimation includes variability from different sources in the laboratory process ensuring better method evaluation (*10*). In our study, all three assays on both analysers have shown acceptable MU with higher values at lower concentrations, since the analytical variability has shown to be the highest at lower concentrations of DOAC.

In this study, we have not done a comparison between global coagulation assays (prothrombin time (PT), activated partial thromboplastin time (aPTT)) and DOACs concentration. Namely, recent studies have reported poor responsiveness of DOACs concentration on global coagulation test results (*11*, *12*). Testa *et al.* have shown that although the correlation between DOACs and screening tests were good, the responsiveness of PT and aPTT was poor, mainly depending on platform and drug (*12*). Prolongation of PT and aPTT has been shown as a concentration dependent in the case of rivaroxaban and dabigatran, whereas results fall within normal ranges in the case of apixaban. Thus, a clinical decision about the anticoagulation effect of DOACs should not be based on the results of screening coagulation tests (PT and aPTT) because it could jeopardize patient safety (*13*).

Due to the lack of LC-MS/MS method, we have not performed verification of trueness and that is the main limitation of the study. However, the presented results are part of the ongoing clinical validation study and the results from both analysers will be observed in that sense. Furthermore, concentrations of the drugs in selected patient samples fell mostly within expected, therapeutic limits with the tendency towards possible under-dosing, thus we did not provide broader ranges of concentrations for method comparison.

In conclusion, chromogenic assays for dabigatran, rivaroxaban, and apixaban concentration determination on STA Compact Max have not met repeatability specifications according to the manufacturer. Additionally, caution must be taken when comparing patient results at lower concentrations of DOACs obtained from BCSXP and STA Compact Max analysers because of the significant bias between methods.
